# THE ROLE OF TEMPORAL CONTEXT IN SHAPING LIFE COURSE TRAJECTORIES: INSIGHTS FROM OLDER CHINESE INDIVIDUALS IN THE UK

**DOI:** 10.1093/geroni/igae098.0440

**Published:** 2024-12-31

**Authors:** Miriam Tenquist

**Affiliations:** University of Manchester, Manchester, England, United Kingdom

## Abstract

International gerontological research has increasingly recognised the importance of larger sociohistorical contexts for understanding the growing needs of an increasingly diverse ageing population. The paper contributes to existing literature by examining how temporal context, including historical time, age cohort, and migration age, influences the life trajectories of older migrant communities, specifically older Chinese migrant communities in the UK. Central to this investigation is an examination of their lived experiences against the backdrop of migration policies and concurrent sociohistorical developments in both the host and home country, both before and during migration. This paper draws on semi-structured interviews with 57 Chinese people over the age of 50 living in the UK more than 5 years. The study identifies two distinct groups within this demographic based on migration waves: the second wave (1945-1980) and the third wave (1980s-2000). The paper highlights disparities in educational opportunities, dispersion patterns, occupation, and socio-linguistic capital between the two waves. Grounded in a life course perspective, this paper emphasises the significance of sociohistorical temporality and spatiality in turning points. We argue that the enduring legacies of Chinese reforms and societal changes are distinctly evident in the life trajectories of the diaspora in the UK, becoming more pronounced in later life stages. Ultimately, the study advocates for a more nuanced understanding of migrant communities that recognise the impacts of distinct migration waves on their unique ageing trajectories. This interdisciplinary, internationally minded perspective allows us to better serve global ageing populations that are increasingly influenced by globalisation.

